# Eco-Friendly Fabrication of 2D a-SnO_x_ Thin-Film Transistors Derived from Deep Eutectic Solvents

**DOI:** 10.3390/ma18235349

**Published:** 2025-11-27

**Authors:** Christophe Avis, Jin Jang

**Affiliations:** Department of Information Display, Advanced Display Research Center, Kyung Hee University, 26, Kyungheedae-ro, Dongdaemun-gu, Seoul 02447, Republic of Korea

**Keywords:** deep eutectic solvent, thin-film transistor, amorphous oxide semiconductor, tin oxide

## Abstract

We have fabricated amorphous tin oxide (a-SnO_x_) thin-film transistors (TFTs) with Al_2_O_3_ gate insulator from deep eutectic solvents (DESs). DESs were formed using the chloride derivates of each precursor (SnCl_2_, or AlCl_3_) mixed with urea. The DESs were then used as precursors for the semiconductor and dielectric. Our target was to form extremely thin semiconductor film, and a sufficient high capacitance insulator. We characterized the physical and chemical properties of the DES-derived thin films by X-ray diffraction (XRD), atomic force microscopy (AFM), and X-ray photoelectron spectroscopy (XPS). We could evaluate that the highest content of metal–oxygen bonds was from the DES condition SnCl_2_–urea = 1:3. At a low 300 °C budget temperature, we could fabricate a 3.2 nm thick a-SnO_x_ layer and 30 nm thick Al_2_O_3_, from which the TFT demonstrated a mobility of 80 ± 17 cm^2^/Vs, threshold voltage of −0.29 ± 0.06 V, and subthreshold swing of 88 ± 11 mV/dec. The proposed process is adequate with the back-end of the line (BEOL) process, but it is also eco-friendly because of the use of DESs.

## 1. Introduction

**Figure 1:**
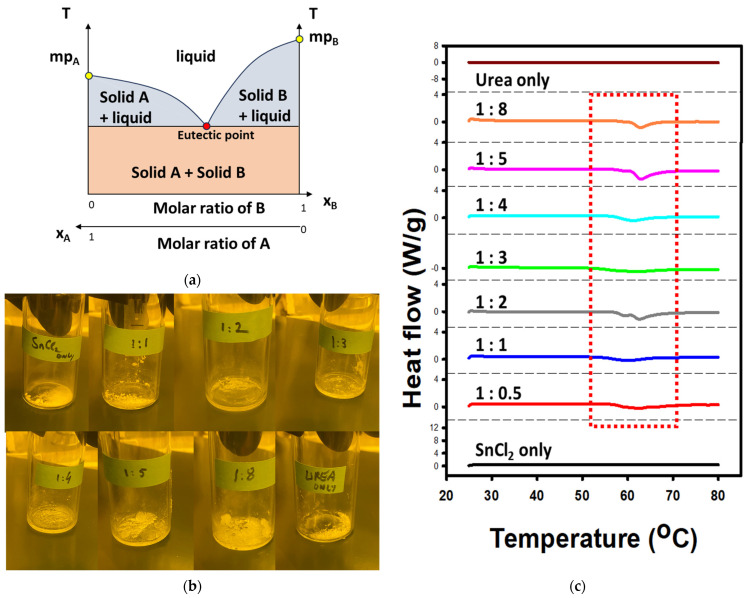
DES formation for mixtures of SnCl_2_–urea: (**a**) General concept of DES formation between two materials, A and B; (**b**) Photos of mixtures of fabricated mixtures of SnCl_2_–urea after being held on a hot plate at 70 °C for 10 min; (**c**) DSC analysis of various SnCl_2_–urea mixtures.

For the past 20 years, deep eutectic solvents (DES) have been studied. Deep eutectic solvents are mixtures of two materials having high melting points (usually above 200 °C) with their mixture having a melting point usually below 100 °C, with examples at room temperature (RT) [1,2]. Figure 1a shows the general concept of DES. When only solid A (B) is heated, the solid phase of A (B) exists until we reach the melting temperature mp_A_ (mp_B_). When we mix together solid A and B, the melting temperature decreases as a function of the ratio of A and B. The minimum melting point is reached at one ratio of A and B, the eutectic. Below that temperature, whatever the ratio, both materials coexist in the solid phase. Above that temperature, for other ratios, a liquid phase and a solid phase coexist until the complete melting occurs.

One of the materials is a hydrogen bond donor (HBD), and the other one is a hydrogen bond acceptor (HBA). The HBD and HBA form hydrogen bonds responsible in part for the formation of a stable DES [1,2,3]. For example, the first reported HBA was choline chloride [1,2]. Other examples are ethylammonium chloride, glycine and so on [2,3,4]. Among HBD, urea was the first one used (with choline chloride), and others are for example acids or alcohols [1,2,3,4]. Contrary to common solvents, DESs are of low toxicity, greener, and can be used multiple times [1,2,3,4,5]. DES properties are high viscosity, low conductivity, and high density solvents. DESs can be used in a wide range of applications. DESs are solvents used in chemistry applications ranging from solvents for various chemical and physical applications [2,3], biology [4,5,6,7], electrodeposition of metals [2,3,4], and even sensors [8]

Thin-film transistors using amorphous oxide semiconductors (AOS) were introduced in 2004 by Nomura et al [9,10]. AOS like indium gallium zinc oxide (IGZO), zinc tin oxide (ZTO), and indium zinc oxide (IZO) have demonstrated their potential use for large-area electronics. These AOS are formed of two cations and one anion, making them usually amorphous. Recently, polycrystalline or crystalline oxide semiconductors have attracted attention due to their potential higher performances in TFTs, and potential use in scaled devices [11,12,13]. In_2_O_3_ and SnO_2_ are the most investigated polycrystalline oxide semiconductors.

Industrial applications like active-matrix organic light-emitting diodes (AMOLEDs) use devices manufactured by vacuum. Sputtering is the most widely employed technique. Recently, In_2_O_3_ has been highly investigated because of its potential use in future devices. Atomic layer deposition (ALD) has been employed to deposit the In_2_O_3_ layer [13]. Along with various doping atoms like Ga, or Sn, In_2_O_3_-based TFTs have reached field-effect mobilities over 50 cm^2^/Vs [14,15,16]. Let us note that various process strategies like CF_4_ plasma treatment [17,18], or various heterojunction channel [19,20]. layers can also help reach high mobilities above 5 m^2^/Vs. In_2_O_3_-based TFTs could even reach mobilities above 100 cm^2^/Vs. The possibility to reach such high mobilities are related to the understanding of the material itself, but also the development of various precursors for ALD.

To compete with the vacuum process, reported solution-processed TFTs have been investigated through two angles. The first one is related to thermal budget, the second one without any consideration of thermal budget. Considering then low temperature process (up to 300 °C), as would vacuum processed ALD or sputtered oxide semiconductor based TFTs, the solution process could reach mobilities of 10 cm^2^/Vs [21,22,23,24,25].

Compared to ALD where many precursors are available and have been developed either “in house” or else [26], the solution process of oxide semiconductors highly suffer from precursor novelties. Main strategies to obtain high performance TFT involve the combustion process [27]. or UV irradiation [28]. Indeed, the main precursors use nitrates as ligands. The combustion process of nitrate precursors can lead to reasonable mobilities of 1–10 cm^2^/Vs at a relatively low process temperature of 300 °C [28,29]. Recently, Quino et al. demonstrated the fabrication of nitrate derivatives out of Cl ligand-based precursors to reach the combustion process [30]. Other strategies involve substitutional doping, high annealing temperatures, double layer channel, or device engineering like dual gate TFT [14,15,16,19,20,31].

Along with vacuum process, solution process has been developed for AOS. Nowadays, spin-coating and spray coating can successfully obtain results in the same order of magnitude as non-vacuum process [10,19,32,33,34,35,36,37,38]. TFTs can reach mobilities over 30 cm^2^/Vs [19,32,33,36,37,38]. The solution process has also lead the path to the use of high-k dielectrics to enhance TFT characteristics [39,40].

Here, we report the use of DESs not as solvents, but as precursors of materials themselves. We propose an opportunity to use DESs for the fabrication of the semiconductor SnO_2_ and the insulator Al_2_O_3_. We then used the semiconductor and the dielectric in thin-film transistors (TFTs) and showed high performance with field-effect mobility of 80 cm^2^/Vs, with 2D amorphous tin oxide (a-SnO_x_) of a thickness of 3.2 nm. The process being kept at 300 °C, the TFTs can be used in further back-end of the line (BEOL) processes, which require a budget temperature of less than 400 °C.

## 2. Materials and Methods

### 2.1. DES and Solution Fabrications

We fabricated precursor solutions of SnO_2_ using DESs of SnCl_2_–urea with various ratios, namely 1:0, 1: 0.5, 1:1, 1:2, 1:3, 1:5, 1:8, where we used 0.5 mmol of SnCl_2_ in each case, and 0, 0,25, 0,5, 1, 1.5, 2.5, and 4 mmol of urea, respectively. First, we mixed the two powders and then placed them on a hot plate at 70 °C for 10 min so that the liquid form appears. Once the liquid phase is formed, we mixed the DES with 25 mL of ethyl acetate and stirred the solution at 70 °C for 1 h.

We also fabricated a precursor solution of Al_2_O_3_ using AlCl_3_–urea with a ratio of 1:1.3 mixed in 5 mL 2-methoxyethanol (2-Me) at RT. The DES appears at RT after 10 min of the mixture of AlCl_3_ and urea.

SnCl_2_ (CAS: 7772-99-8, purity 99.99%), urea (CAS: 57-13-6, purity: 99%), 2-Me (CAS: 109-86-4, purity: 99.8%), and ethyl acetate (CAS: 141-78-6, purity: ≥ 99.5%) were all purchased from Sigma-Aldrich (Yongin-Si, Republic of Korea). AlCl_3_ (CAS: 7446-70-0, purity: 99.999%) was manufactured by Thermo Fisher scientific (Ward Hill, MA, USA).

### 2.2. Thin-Film Transistor Fabrication and Evaluation

Thin-film transistors were fabricated by first depositing Mo (40 nm) by sputtering on glass. After patterning the metal as the gate, we spin-coated the Al_2_O_3_ precursor solution, cured at 150 °C for 5 min, and at 300 °C for 5 min. The step was repeated once to obtain a 30 nm thick Al_2_O_3_ layer. The Al_2_O_3_ layer was left to anneal on a hot plate at 300 °C for 2 h. We then spin-coated the SnO_2_ precursor solution and followed the same curing step as for the Al_2_O_3_ solution. After patterning and annealing at 300 °C for 2 h, via holes were formed and a 200 nm IZO layer was sputtered, and it was patterned as the source and drain (S/D). The final product was annealed at 300 °C for 6 h. Note that all patterning steps were performed by common photolithography.

We performed IV curve measurements on a 4156C Agilent semiconductor parameter analyzer (Agilent Technologies, Santa Clara, CA, USA). From the measurement, we extracted the subthreshold swing (SS) as $\frac{\partial V_{GS}}{\partial log(I_{DS})}$, the V_th_ defined as W/L × 10 ^− 10^A, and the field-effect mobility µ_FE_ defined as $\frac{\partial I_{DS}}{\partial V_{GS}}\frac{L}{WC_{ox}V_{DS}},$ where W, L, C_ox_, V_DS_, V_GS_, and I_DS_ are the width of the TFT, the length of the TFT, the capacitance of the insulator, the applied drain voltage, and the applied gated voltage, respectively [41,42].

We measured the capacitance–frequency (C-f) characteristics of the Al_2_O_3_ layer on a MIM structure (M = Mo, 40 nm, I: Al_2_O_3_, 30 nm) with an Agilent E4980A precision LCR meter (Agilent Technologies, Inc., Santa Clara, CA, USA). The TFTs had a width and length of 50 and 10 µm, respectively. The average values given in the text are from 30 TFTs.

### 2.3. DES and Thin-Film Analysis

The deep eutectic solvents (DESs) were analyzed by differential scanning calorimetry using a DSC-250 instrument (TA Instruments, New Castle, DE, USA). The mixtures were analyzed by increasing the temperature from room temperature to 80 °C. Surface roughness was analyzed by atomic force microscopy (AFM) using a XE-7 system (Park Systems, Suwon, South Korea) with a tapping method on a 5 μm × 5 μm area to obtain the root mean square roughness (RMS roughness). The O1s spectrum of SnO_2_ was analyzed by X-ray photoelectron spectroscopy (XPS) using a Phi 5000 Versaprobe system (ULVAC-PHI, Chigasaki, Kanagawa, Japan), calibrated at the 284.6 eV C1s peak. For depth profile measurements, the etching was performed by Argon ions at 2 kV with a sputter rate of 24 nm/min.

The crystallinity properties of the layers were evaluated by X-ray diffraction (XRD) (Rigaku, Tokyo, Japan). The Cu-Kα radiation (λ = 1.54 Å) was used for XRD measurements. Note that for XRD measurements, the thin films were 30–40 nm thick. Transmission electron microscopy (TEM) images were obtained using a Titan 80–300 microscope (Thermo Scientific™, former FEI, Hillsboro, OR, USA). The accelerating voltage was 300 kV, the detector was a OneView 1094 camera (Gatan, Inc., Pleasanton, CA, USA), and the vacuum level was 10^−7^ mbar.

## 3. Results and Discussions

### 3.1. DES Analysis

Figure 1b shows the various mixtures of SnCl_2_–urea at a temperature of 70 °C. Some mixtures appear formed of a liquid and a solid phase, while only the 1:3 ratio demonstrates a liquid phase, namely the eutectic point. The transparent and liquid form of the mixture demonstrate the formation of the DES. Let us note that the name DES is commonly used for any ratio [43,44].

We demonstrate the apparition of the liquid phase of the DES and other ratios under heat by differential scanning calorimetry (DSC) as shown in Figure 1c. We observed the formation of the liquid by the endothermic peak by ~55–65 300 °C for SnCl_2_–urea mixtures. Urea only and SnCl_2_ only do not show this peak. This confirms the apparition of the liquid phase of the DES in Figure 1b when the mixtures are heated at 70 °C. Note that other reports on the fabrication of DES use a temperature up to 80 °C because higher temperature would degrade the DES [1,2,3,4,5].

### 3.2. Thin-Film Analysis Results and Discussions

**Figure 2:**
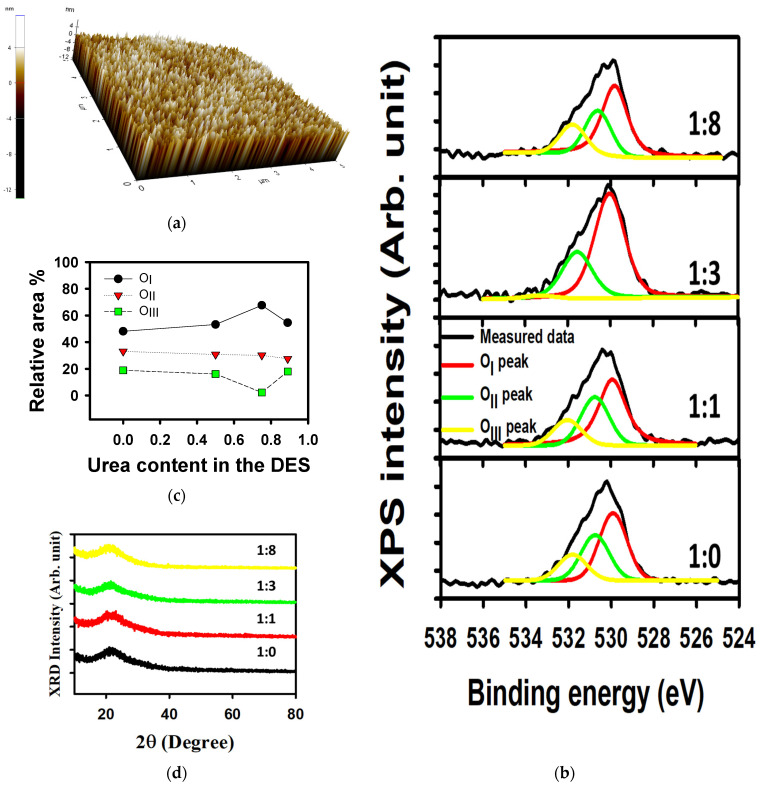
Analysis of DES-derived tin oxide thin films. (**a**) AFM image of the thin film derived from the SnCl_2_–urea 1:3 DES, (**b**) XPS O1s peak deconvolution, (**c**) summary of the deconvolution, and (**d**) XRD spectrum of the thin films made from SnCl_2_–urea = 1:0, 1:1, 1:3, and 1:8.

An important feature of a thin film is the surface morphology. We analyzed the surface by AFM on 5 μm × 5 µm sized samples. The AFM image of the thin film made from the solution with the DES composition (SnCl_2_–urea = 1:3) is shown in Figure 2a. Thin films made with other compositions are shown in Supplementary Figure S1. When increasing the SnCl_2_–urea ratio from 1:0 to 1:1, 1:3, and to 1:8, the root mean square (RMS) roughness of the layer varied from 1.705, to 1.643, 2.307, and to 2.038 nm, respectively. These RMS roughnesses are rather smooth and acceptable for microelectronic applications. Isakov et al. analyzed their In_2_O_3_ thin film made by spray pyrolysis with conventional nitrate precursors, and observed that the thinner their channel layer was, the rougher their surface was [45].

To have an insight into the thin-film compositions, we analyzed the O1s spectrum measured by X-ray photo-spectroscopy (XPS). The conventional analysis of the peak corresponds to a deconvolution of the contour into three main peaks: O_I_ related to metal–oxygen (O-M) bonds, O_II_ related to oxygen vacancies, and O_III_ related to -OH groups. Specifically, from SnCl_2_–urea = 1:0, 1:1, 1:3, and 1:8 O_I_ ratios were 48.12, 53.20, 67.62, and 54.54%, respectively; O_II_ ratios were 33.04, 30.70, 30.12, and 27.52%; and O_III_ ratios were 18.84, 16.10, 2.26, and 17.94%, respectively. The deconvoluted O1s peaks for films with various SnCl_2_–urea ratios are shown in Figure 2b. Therefore, the amount of M-O bonds (-OH bonds) is greatest (smallest) for the DES condition of SnCl_2_–urea 1:3. A summary of the various peak relative content in the films are shown in Figure 2c. Let us note that the carbon-related peak was present on the surface of each sample but not present inside the films as shown in Supplementary Figure S2. Reliability of the XPS experiment is necessary to be emphasized, as the resolution of the measurement system is ±0.5%. So, even though the XPS measurement demonstrates the absence of carbon, or other contaminants, let us keep in mind that the accuracy is for 1 atom per 200. Higher resolution measurement could be used, like time-of-flight secondary ion measurement spectrometry (TOF-SIMS) [38].

The crystallinity was investigated for the thin films fabricated from the SnCl_2_–urea = 1:0, 1:1, 1:3, and 1:8 by X-ray diffraction (XRD). The results are shown in Figure 2d. All films show an amorphous phase, consistent with previous work using SnCl_2_ as a precursor of tin oxide [38]. The observed broad peak is related to the amorphous phase of the glass substrate [9].

**Figure 3:**
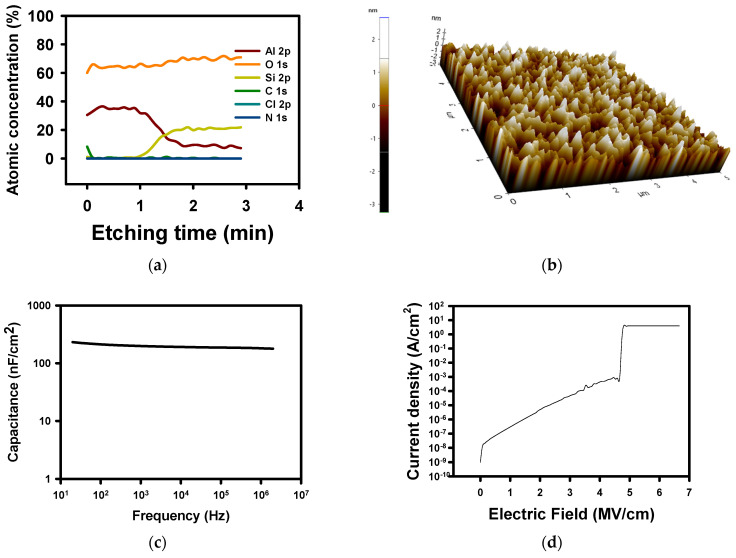
Analysis of the Al_2_O_3_ thin film made from AlCl_3_–urea = 1:1.3 DES. (**a**) XPS depth profile of the Al_2_O_3_ layer, (**b**) AFM image, (**c**) C-f characteristics, and (**d**) current density as a function of the voltage applied to determine the breakdown voltage.

Following the successful fabrication of SnO_2_ thin films, we decided to investigate the properties of a DES-processed high-k dielectric. For this purpose, we demonstrate the possibility to make Al_2_O_3_ dielectric by a DES approach. We used a AlCl_3_–urea ratio of 1:1.3. (cf Supplementary Figure S3). The DES is obtained at RT, and no further heating is necessary. Let us note that other groups reported DES of AlCl_3_–urea for other applications like batteries [46,47,48]. We fixed the ratio at 1:1.3 as the purpose is to demonstrate the feasibility of fabrication of Al_2_O_3_ rather than an optimization. Figure 3a shows the XPS depth profile of the DES-derived Al_2_O_3_ thin film. The figure shows that C is present at the surface and not in the thin film. There is also no residue of N or Cl in the thin film, demonstrating that the DES-derived thin film leads to no residues or carbon contamination. As a decrease in Al content appears, the Si content increases. The Si peak comes from the glass substrate. The XPS measurement beam provides information not only on the surface atoms, but for a thickness of 10 nm. Therefore, we cannot see any abrupt variation in any atomic concentration. From the depth profile, we can understand the thickness of the Al_2_O_3_ layer is 30 nm. We could obtain a 30 nm Al_2_O_3_ thin film having an RMS roughness of 0.705 nm as shown in Figure 3b. We measured the capacitance characteristics of the dielectric from a MIM structure (cf. Figure 3c). The breakdown voltage measured for the Al_2_O_3_ dielectric was 4.81 ± 0.25 MV/cm (cf. Figure 3d), and a k value of 7.62 ± 2.16 (corresponding to a capacitance value of 225 ± 63.8 nF/cm^2^ at 20 Hz) close to the theoretical value of 9 [49]. Also, let us note the current density of 2.7 10^−7^ A/cm^2^ at 1 MV/cm. The results are largely better than the previously reported Al_2_O_3_ manufactured by solution processes with AlCl_3_ [34].

### 3.3. TFTs Results

**Figure 4:**
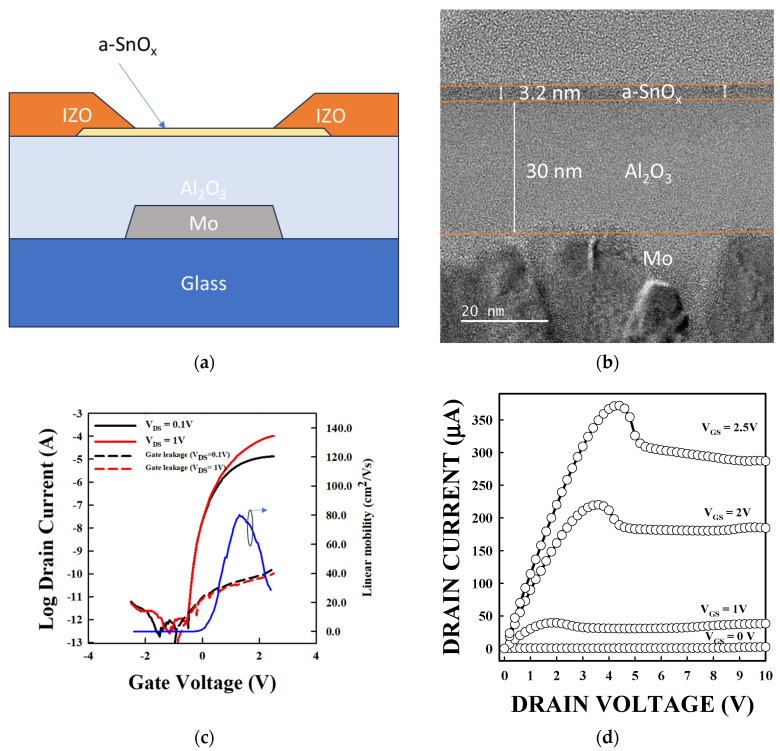
Characteristics of a-SnO_x_ TFT made from DES of SnCl_2_–urea = 1:3. (**a**) TFT structure, (**b**)TEM image, (**c**) transfer curve, and (**d**) output curve.

We used the Al_2_O_3_ and SnO_2_ layers to manufacture TFTs. Figure 4a shows a schematic of the TFT structure. Figure 4b shows a TEM image of a TFT made with DES-derived Al_2_O_3_ and DES-derived SnO_2_ (SnCl_2_–urea = 1:3). The image demonstrates a DES-derived Al_2_O_3_ of 30 nm, and a DES-derived a-SnO_x_ of 3.2 nm. The image clearly demonstrates the possibility of fabricating an extremely thin semiconductor layer of a-SnO_x_. With SnCl_2_–urea ratio varying from 1:0 to 1:1, 1:3, and to 1:8, we obtained mobilities of 54 ± 10, 61 ± 8, 80± 17, 75 ± 15 cm^2^/Vs, V_th_ of −0.14 ± 0.02, −0.19 ± 0.04, −0.29 ± 0.06, and −0.25 ± 0.05 V, and SS of 86 ± 4, 90 ± 4, 88 ± 11, and 92 ± 10 mV/dec., respectively. The TFT with the highest performances is obtained at the DES condition (SnCl_2_–urea = 1:3). The mobility trend follows the M-O-M density revealed by the analysis of the O1s peak. It is understood that a higher overlap of the cation orbitals is therefore an indication of the higher current flow of the electrons leading to a higher mobility in the channel layer made from SnCl_2_–urea = 1:3 [9,10].

Figure 4c,d show the current–voltage (I–V) transfer and output curves of a TFT made from the SnCl_2_–urea = 1:3 DES condition, respectively. Supplementary Figure S4 shows the transfer and output curves of TFTs with a-SnO_x_ made from SnCl_2_–urea = 1:0, 1:1, and 1:8. Let us note that even though the output shows saturation, a local maximum is present (by V_DS_ = 4 V). This could be due to a local heating at/near the S/D due to the high current, as was also reported before in IGZO TFTs [50]. So further engineering of the S/D is necessary. Let us note that in terms of S/D engineering, Lin et al. demonstrated that the gate metal has an impact on the V_th_, as, physically, the metal can create oxygen bonds, leading to oxygen vacancies in the channel [51].

In_2_O_3_ has been widely investigated compared to SnO_2_, and we will first discuss results of In_2_O_3_ TFTs. Processes like spray pyrolysis or ALD can also lead to thin layers, but require high temperatures, dual-gate TFT structures, or heterostructures as the channel layer. For example, a thin In_2_O_3_ below a thick ZnO can lead to a 2D electron gas (2DEG) layer with potentially high mobilities but require a thermal budget over 300 °C [19,35]. Spray pyrolysis of In_2_O_3_ demonstrated confinement effect and that the conduction of electrons is limited by the roughness of the (extremely) thin layer [45]. Conventionally, ALD is used to fabricate high-quality and smooth layers, for example, In_2_O_3_ thicknesses as low as 0.7 nm [13]. Our process clearly demonstrates the potential use of DES for the fabrication of a-SnO_x_ leading to high-quality TFTs. SnO_2_ (or a-SnO_x_) has shown less interest. Comparatively, tin oxide thin films made by the solution process have a higher thermal budget than ours (usually at least 350 °C). G. Huang et al. fabricated SnO_2_ TFT on Al_2_O_3_ at 350 °C to reach a mobility of 96.4 cm^2^/Vs, V_th_ of 1.72 V, and SS of 0.26 V/dec., but they did not report the stability of their TFTs [36]. Shih et al. also reported mobility reaching 147.5 cm^2^/Vs for an annealing temperature of 400 °C [37].

**Figure 5:**
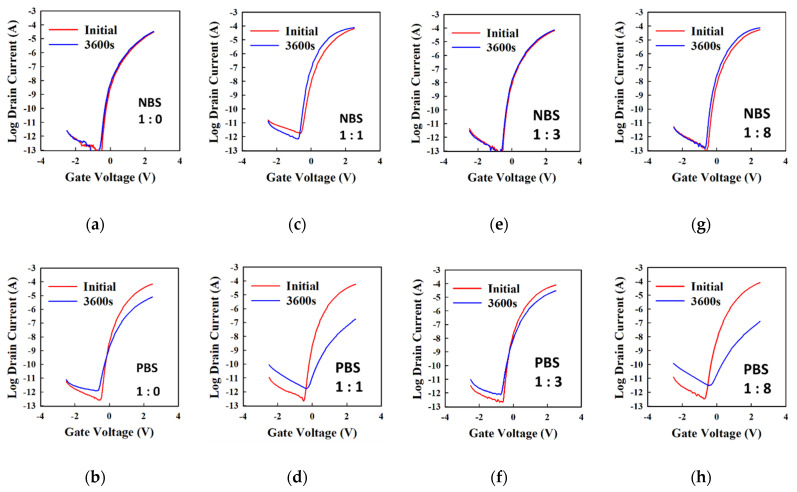
PBS and NBS results of SnO_2_ TFT with Al_2_O_3_ and SnO_2_ made by DES. (**a**,**b**), (**c**,**d**), (**e**,**f**), and (**g**,**h**) represent the transfer curves before and after NBS and PBS, respectively, for the TFT using a-SnO_x_ made from SnCl_2_–urea = 1:0, 1:1, 1:3, and 1:8. The red (blue) curve represents the IV transfer curve measured at the beginning (end) of the stress.

We also tested the TFT under conventional positive bias stress (PBS) and negative bias stress (NBS). PBS (NBS) was performed by applying + 2.5 V (−2.5 V) at the gate for 1 h. The results are shown below in Figure 5. Even though there is no significant shift under PBS, we do observe a decrease in the total current after 1 h of PBS, and a decrease in the OFF current. An increase in the OFF current reveals that the number of electrons available in the negative V_GS_ region is higher. The slight increase in the SS demonstrates the creation of defect states. The defect states are usually oxygen vacancies, freeing electrons in the channel [9], but the number of defects can become too high, and because of scattering, the channel layer does not conduct electrons efficiently, leading to the reduction in ON current. The decrease in ON current is a problem observed in a previously reported a-SnO_x_ TFT [38]. Under NBS, we do not observe a significant shift for the TFT with the DES condition (1:3) and for the TFT made from Sncl_2_ only. Therefore, urea can be introduced to enhance the TFT initial characteristics at exactly the 1:3 DES condition, and other SnCl_2_–urea leads to degradation of the TFT under PBS and NBS because of the creation of defects. Since the TFT stability does not relate to the a-SnOx oxygen environment, lower state creation for the TFT using SnCl_2_–urea of 1:0 and 1:3 is more related to the gate insulator/SnO_2_ interface than the SnO_2_ material itself.

Finally, we want to discuss the opportunity to fabricate new precursors for oxide semiconductors. Even though solution process has demonstrated many competitive results compared to the industry-preferred vacuum process, there has been an evident lack in new processes and precursors. It is easy to note by reading state-of-the-art solution-processed TFTs that the precursors employed are usually nitrates (NO_3_)^−^. State-of-the-art solution-processed oxide TFTs made from nitrate precursors can hardly reach 10 cm^2^/Vs at 300 °C, but can reach 20 cm^2^/Vs using combustion processes or other strategies like radiation [22,23,25,32,33,52]. Chloride precursors, on the other hand, are usually considered to be corrosive, and have been investigated by fewer numbers of researchers. Used in IGO (indium chloride as the precursor for indium), they can lead to TFTs having mobilities around 30 cm^2^/Vs [39,53]. Let us note the use of tin (II) 2-ethylhexanoate by Huang et al. to obtain high-performance SnO_2_ TFTs [36]. Nonetheless, there is an evident lack of new precursors, which is important in any semiconductor fabrication. As for ALD, many precursors have been developed for In_2_O_3_ [11], and the understanding of the relationship between precursor and TFT result is paramount. So, our strategy tries to participate in solving this problem and fabricate new precursors via the use of deep eutectic solvents serving as precursors, even though we did not remove the chloride ligands.

## 4. Conclusions

We fabricated DES, not to be used as solvents, but to manufacture thin films. DESs of SnCl_2_–urea and DESs of AlCl_3_–urea were used to make the semiconductor a-SnO_x_ and the dielectric Al_2_O_3_. The method employs a thermal budget of 300 °C and can lead to high-quality thin films. The a-SnO_x_ films are smooth and amorphous, and the DES (SnCl_2_–urea = 1:3)-derived thin films leads to the film with the higher density of O-M bonds. The Al_2_O_3_ derived from AlCl_3_–urea DESs demonstrated a smooth surface, and without N, Cl, and C residues that could have come from either the solvent or urea. As a dielectric, Al_2_O_3_ demonstrated a breakdown voltage of 4.65 MV/cm, and a k value of 7.62. We fabricated TFTs using both layers and obtained mobilities of 80 cm^2^/Vs, for the DES condition of SnCl_2_–urea = 1:3, and AlCl_3_–urea = 1:1.3. The TFTs show adequate responses under PBS and NBS. DES-derived thin films are an eco-friendly method to manufacture 2D a-SnO_x_ and Al_2_O_3_ thin films for TFTs. They can also be applied to BEOL applications.

## Data Availability

The original contributions presented in this study are included in the article/Supplementary Material. Further inquiries can be directed to the corresponding author.

## Supplementary Materials

The following supporting information can be downloaded at https://www.mdpi.com/article/10.3390/ma18235349/s1; Figure S1: AFM images of SnCl_2_–urea-derived thin films; Figure S2: C1s peak presence in films derived from various SnCl_2_–urea ratios, Figure S3: DES of a mixture of AlCl_3_–urea; Figure S4: TFT characteristics derived from various SnCl_2_–urea ratios.
