# Supplementary material for: Eco-Friendly Fabrication of 2D a-SnOx Thin-Film Transistors Derived from Deep Eutectic Solvents
Source: Materials (Basel). 2025 Nov 27;18(23):5349. doi: 10.3390/ma18235349 (PMC12693035; doi:10.3390/ma18235349)
Supplement: Supplementary file 1 [file materials-18-05349-s001.zip › materials-3962307-supplementary.pdf]

Supporting Information for

Eco-friendly Fabrication of 2D a-SnO_x Thin-Film Transistors Derived from Deep Eutectic Solvents

By

Christophe Avis and Jin Jang

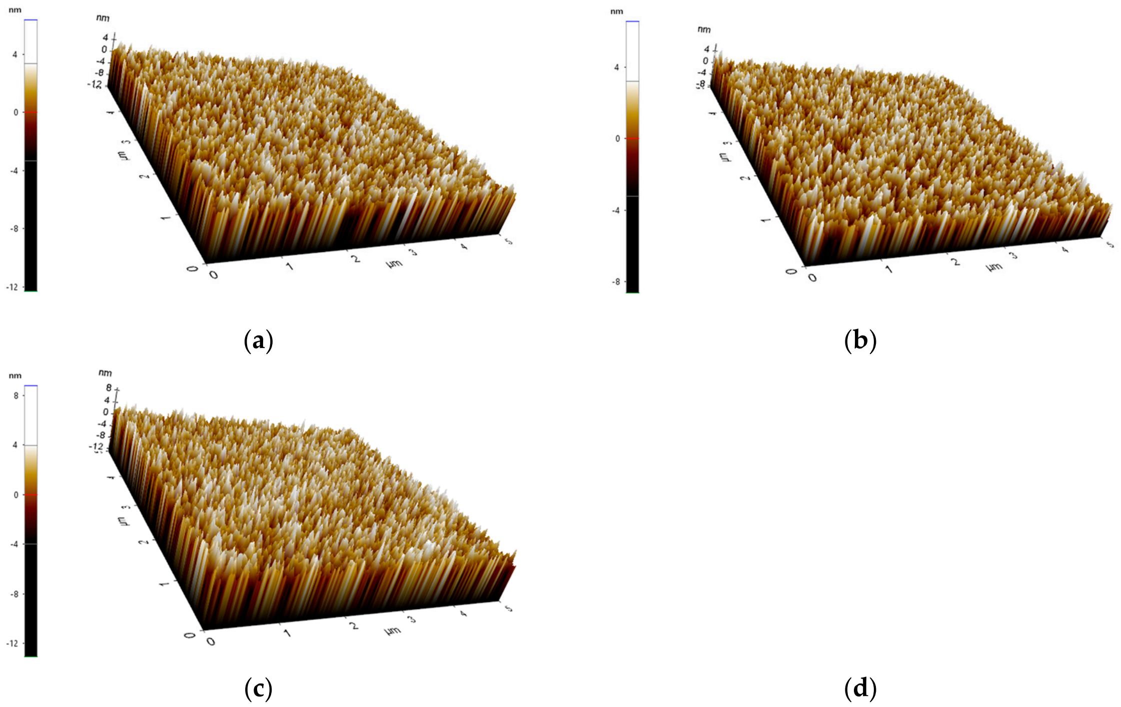

Figure S1. AFM images of $SnCl_2$–urea with ratios of (a) 1:0, (b) 1:1, and (c) 1:8.

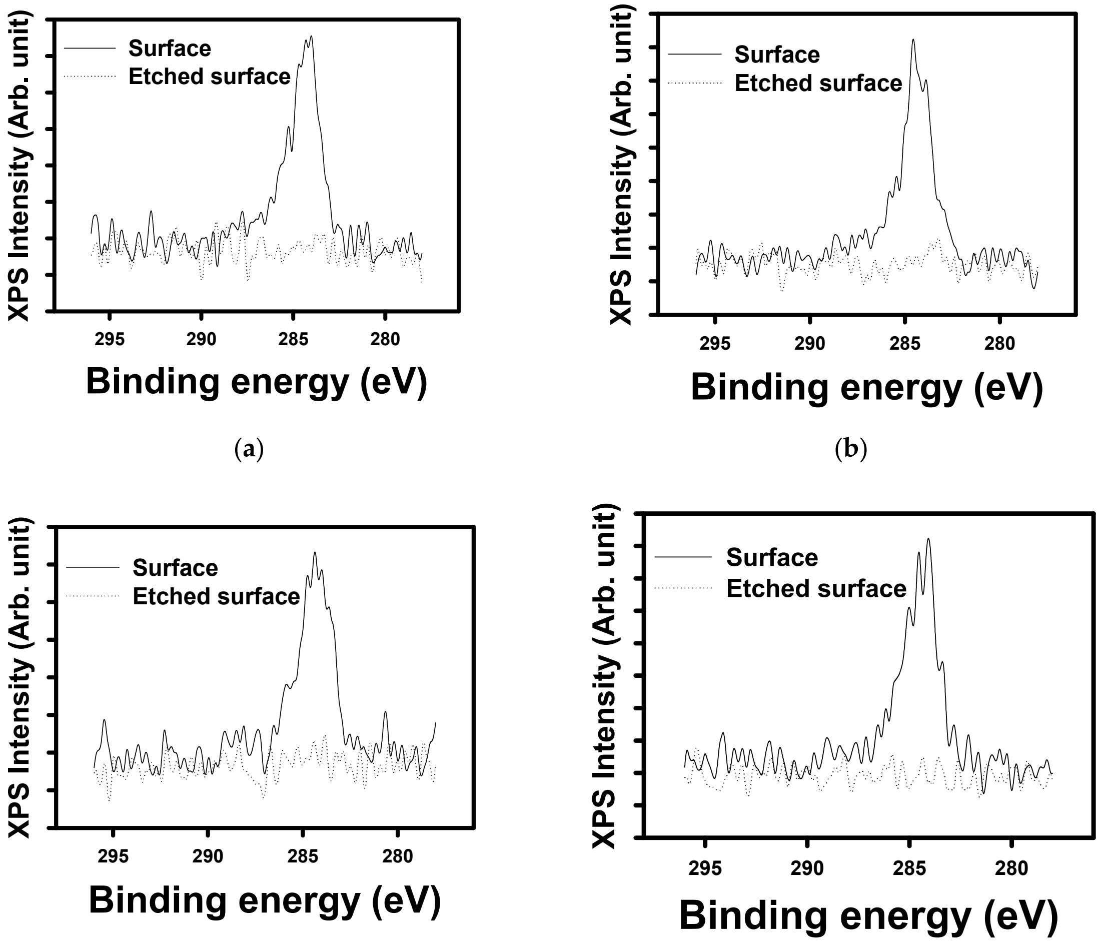

Figure S2. C1s peak measured by XPS at the surface and on an etched surface of a thin film of a-SnO_x made from a $SnCl_2$–urea ratio of (a) 1:0, (b) 1:1, (c) 1:3, and (d) 1:8.

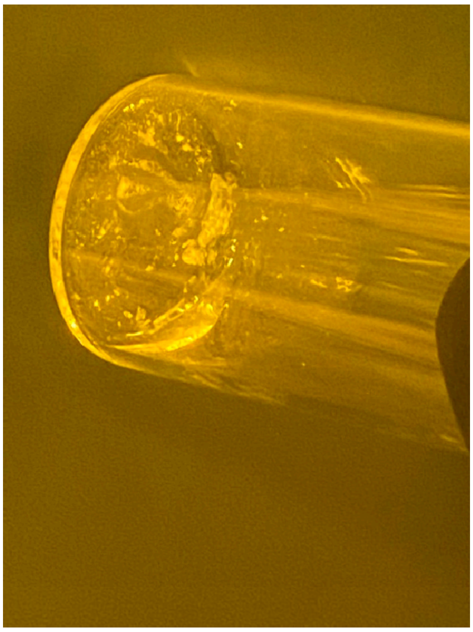

Figure S3 DES of a mixture of $AlCl_3$–urea. We can observe the solid and liquid phase of such a mixture.

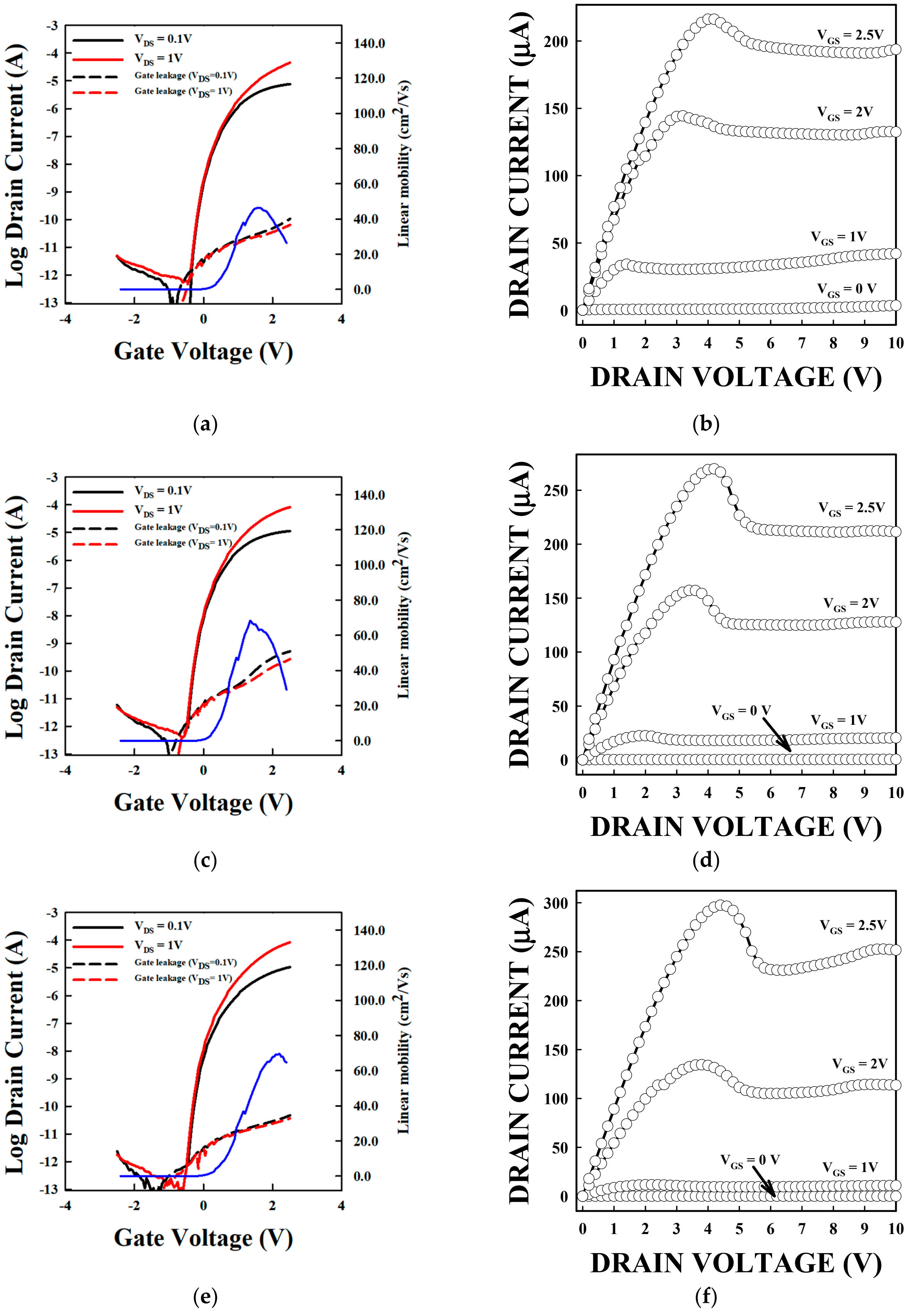

Figure S4. (a) and (b), (c) and (d), and (e) and (f) are the typical TFT transfer and output characteristics of a-SnO_x made from $SnCl_2$–urea with ratios of 1:0, 1:1, and 1:8, respectively.
